# Supplementary material for: MEMO: Mass Spectrometry-Based Sample Vectorization to Explore Chemodiverse Datasets
Source: Front Bioinform. 2022 Apr 13;2:842964. doi: 10.3389/fbinf.2022.842964 (PMC9580960; doi:10.3389/fbinf.2022.842964)
Supplement: Supplementary file 1 [file Table1.DOCX]

Supplementary Material

## Supplementary Figures

####
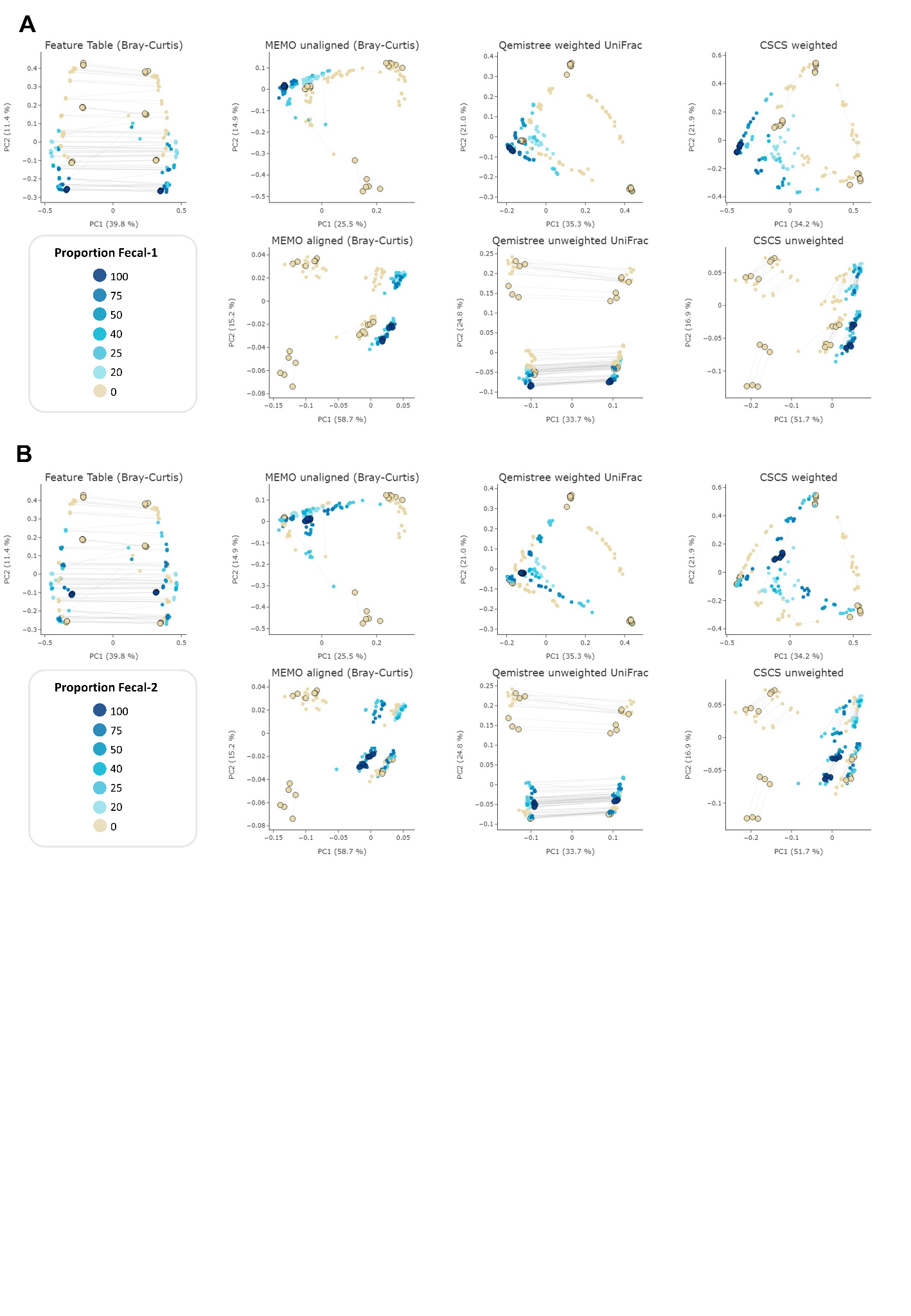


**Supplementary Figure 1.** PCoA comparison of a classical and MS/MS agnostic approach (Feature Table Bray Curtis) and three MSMS informed clustering approaches (the MEMO from unaligned/aligned (Bray-Curtis distance), Qemistree weighted/unweighted (UniFrac distance), weighted/unweighted CSCS and Feature-Table (Bray-Curtis distance) clustering) on the evaluation dataset acquired using 2 different LC methods on the Q-Exactive mass spectrometer (C18 and C18 RT-shift). Samples are colored according to the proportion of fecal-1 sample (**A**) or fecal-1 sample (**B**). For statistical analysis, see Table 1. The samples corresponding to the same mixture and replicate in each dataset (C18 and C18 RT-shift) are linked (gray line). Parent samples are bigger and have a black border line. Interactive visualizations are available at <https://mandelbrot-project.github.io/memo_publication_examples/>.


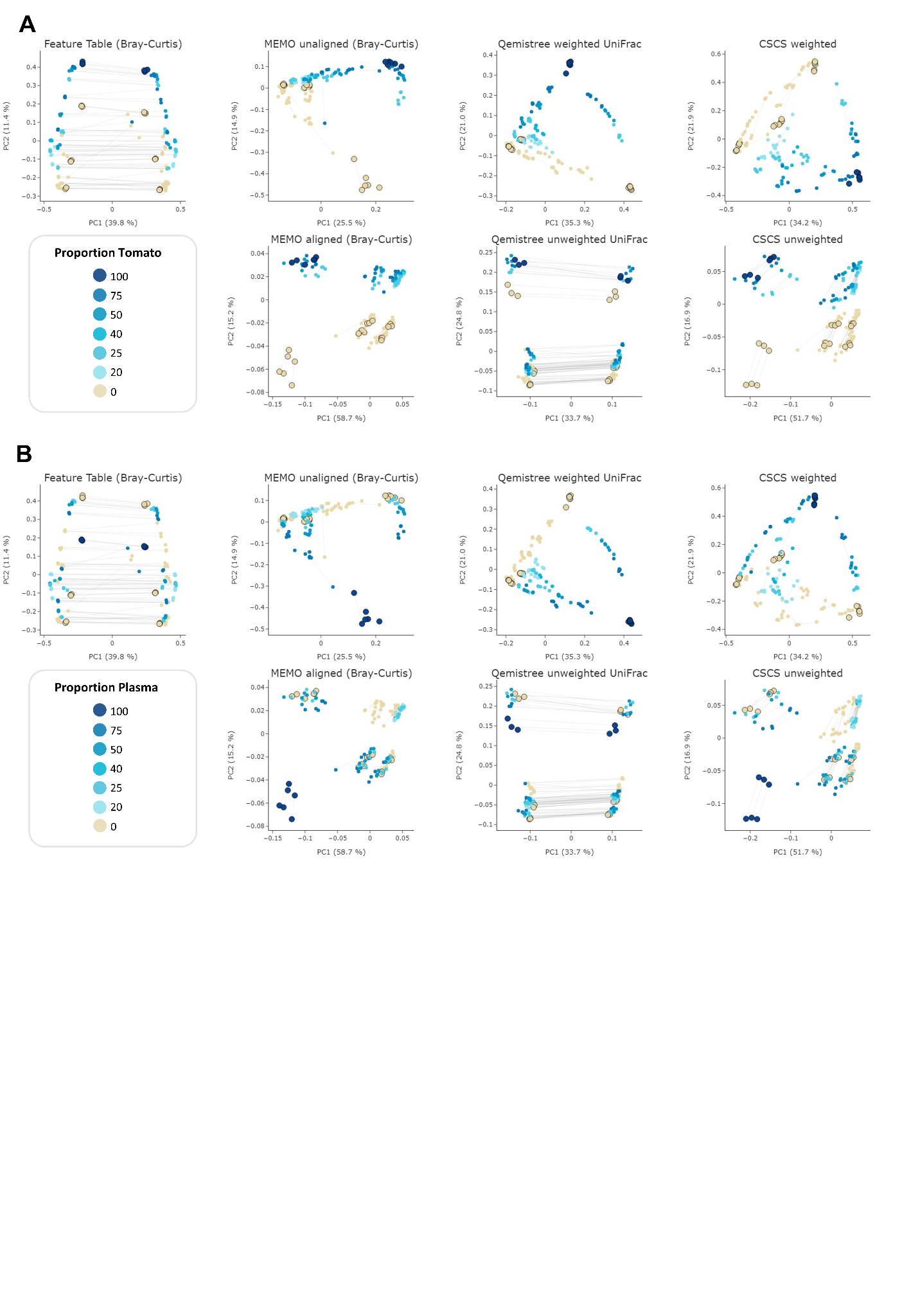


**Supplementary Figure 2.** PCoA comparison of a classical and MS/MS agnostic approach (Feature Table Bray Curtis) and three MSMS informed clustering approaches (the MEMO from unaligned/aligned (Bray-Curtis distance), Qemistree weighted/unweighted (UniFrac distance), weighted/unweighted CSCS and Feature-Table (Bray-Curtis distance) clustering) on the evaluation dataset acquired using 2 different LC methods on the Q-Exactive mass spectrometer (C18 and C18 RT-shift). Samples are colored according to the proportion of tomato sample (**A**) or plasma sample (**B**). For statistical analysis, see Table 1. The samples corresponding to the same mixture and replicate in each dataset (C18 and C18 RT-shift) are linked (gray line). Parent samples are bigger and have a black border line. Interactive visualizations are available at <https://mandelbrot-project.github.io/memo_publication_examples/>.


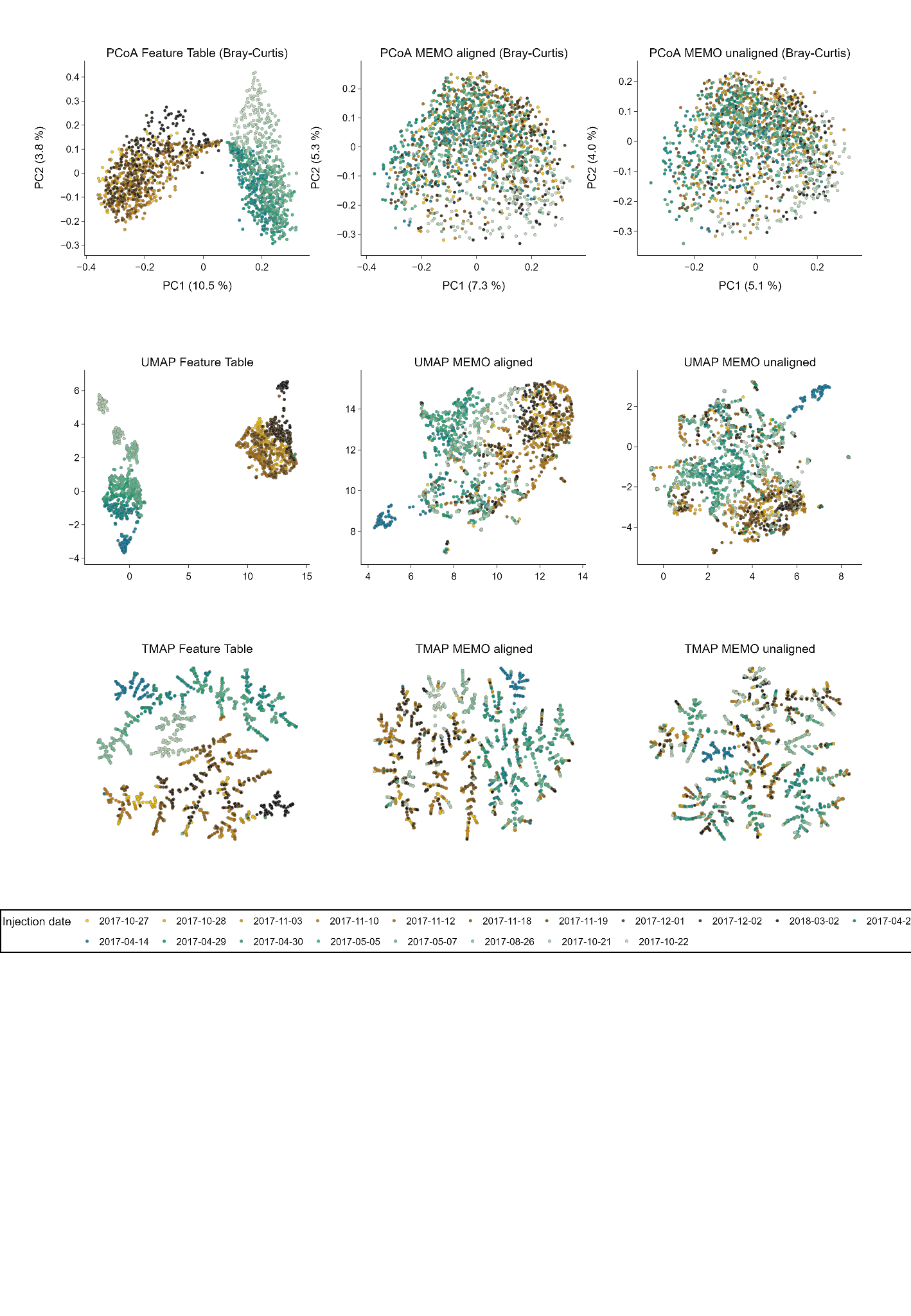


**Supplementary Figure 3.** PCoA, UMAP and TMAP visualizations of the Feature Table, MEMO from aligned and MEMO from unaligned matrices of the plant extract dataset (n=1600) with samples colored according to their injection date. Interactive visualizations are available at <https://mandelbrot-project.github.io/memo_publication_examples/>.


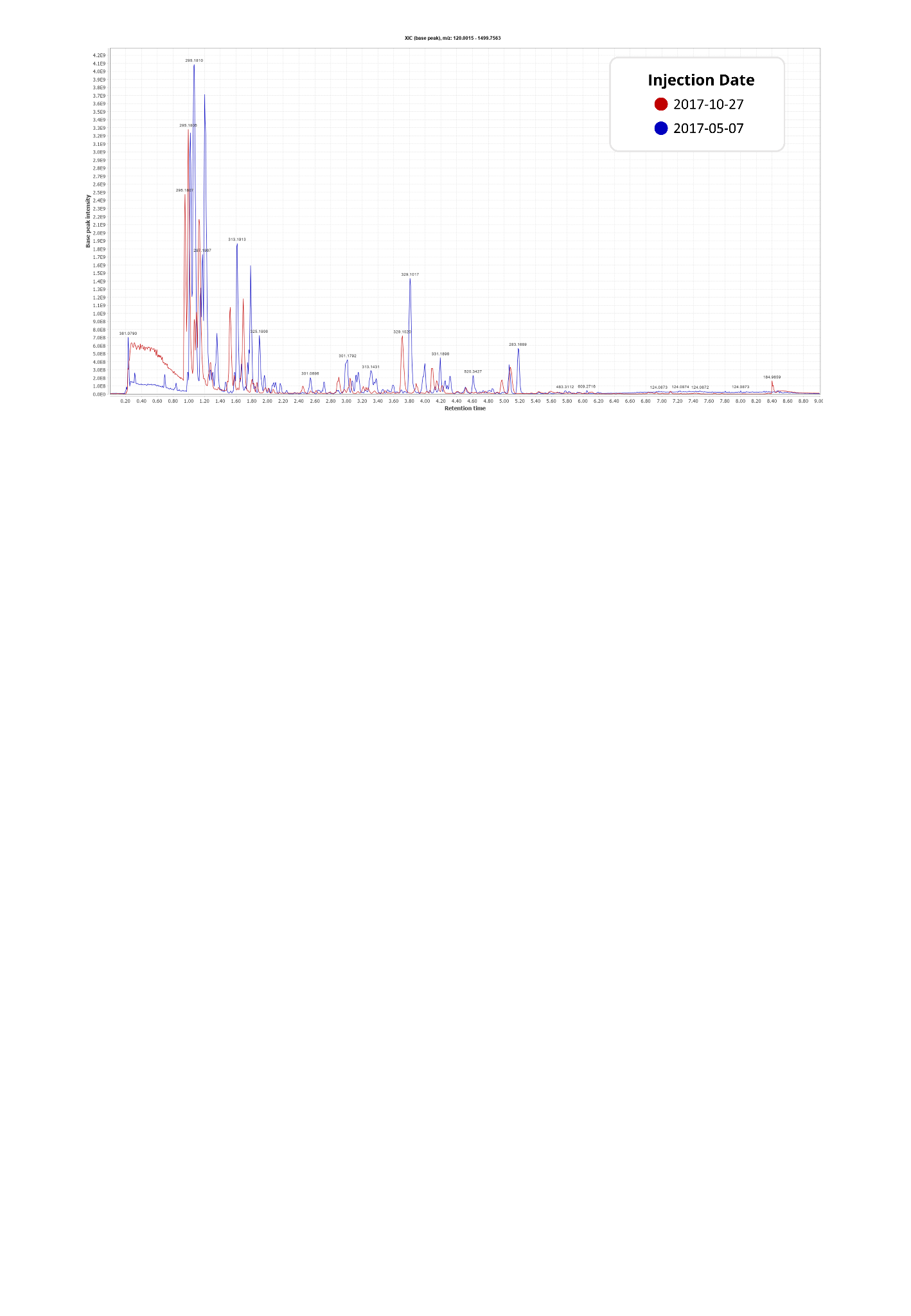


**Supplementary Figure 4.** TIC ESI-(+) of two representative QC samples from each of the two observed batches. QC sample is a mixture of 5 plant ethyl acetate extracts (*Arnica montana*, *Cinchona succirubra* (syn. *Cinchona pubescens*), *Ginkgo biloba*, *Panax ginseng*, *Salvia officinalis*).


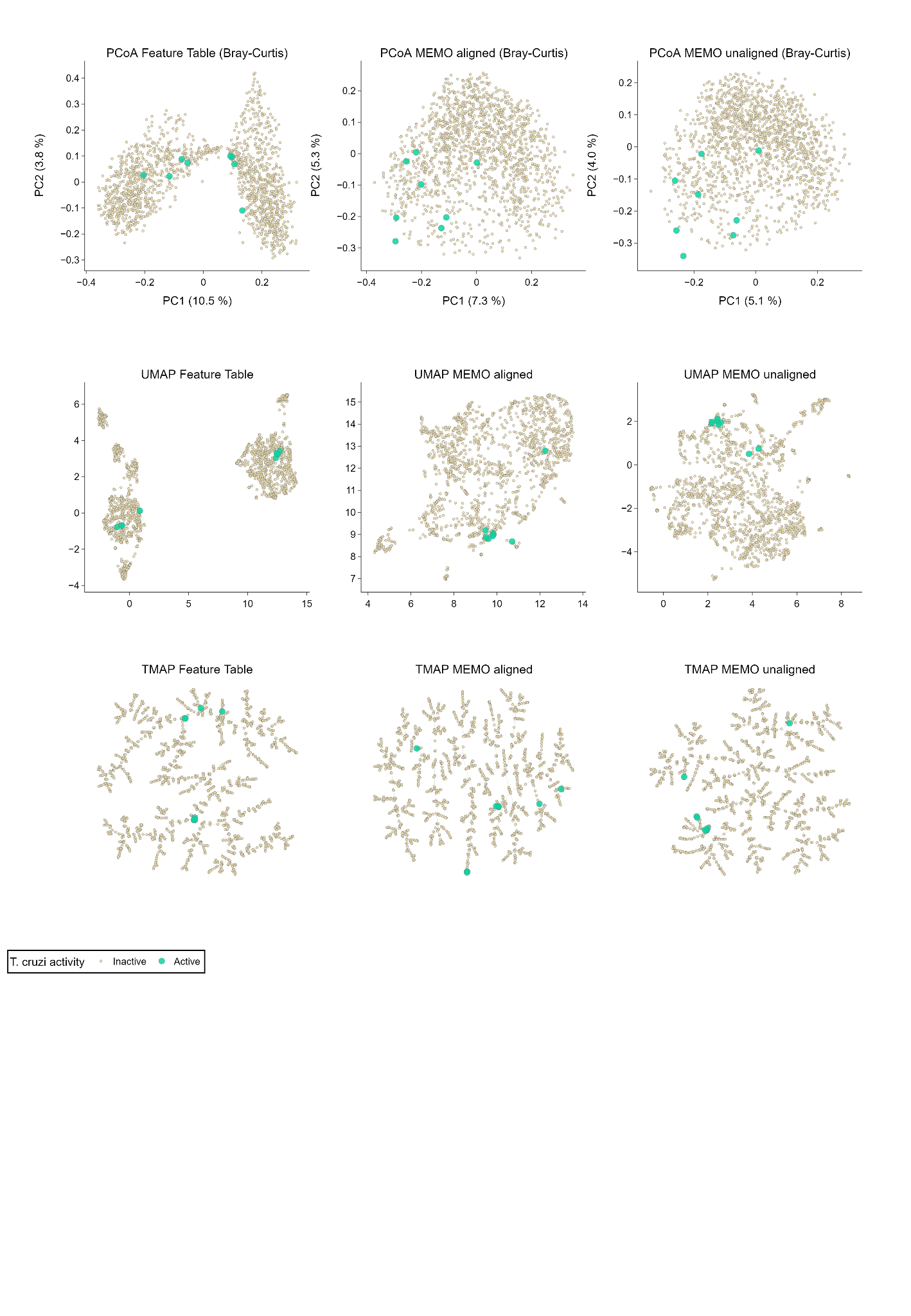


**Supplementary Figure 5.** PCoA, UMAP and TMAP visualizations of the Feature Table, MEMO from aligned and MEMO from unaligned matrices of the plant extract dataset (n=1600) with samples colored according to their activity against *Trypanosoma cruzi*. Interactive visualizations are available at <https://mandelbrot-project.github.io/memo_publication_examples/>.


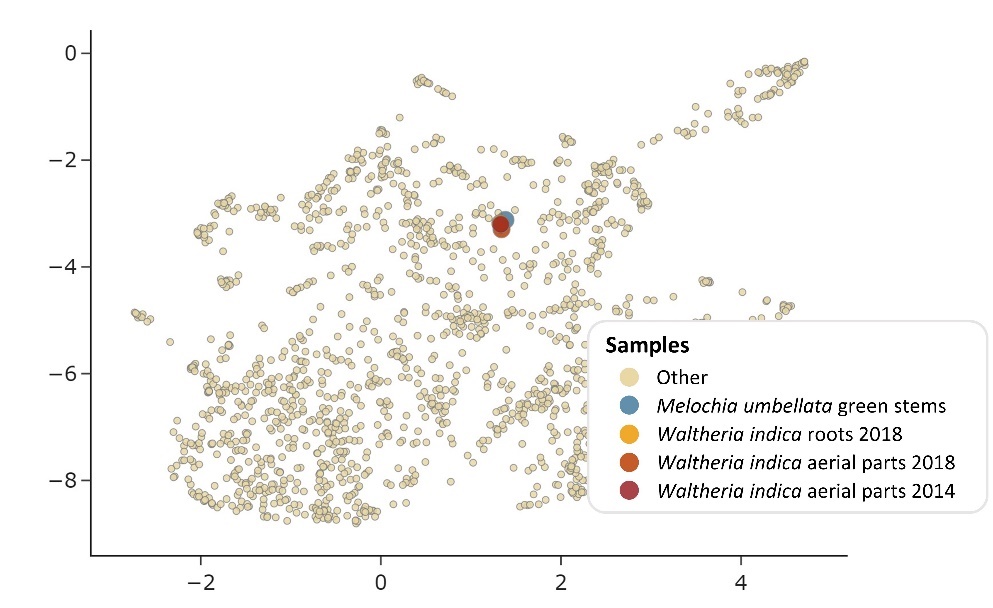


**Supplementary Figure 6.** UMAP visualization of the MEMO from unaligned samples of the plant extract dataset along with three waltherione-rich extract samples from *Waltheria indica* (n=1603 samples). Interactive visualizations are available at <https://mandelbrot-project.github.io/memo_publication_examples/>.
