# Supplementary material for: MEMO: Mass Spectrometry-Based Sample Vectorization to Explore Chemodiverse Datasets
Source: Front Bioinform. 2022 Apr 13;2:842964. doi: 10.3389/fbinf.2022.842964 (PMC9580960; doi:10.3389/fbinf.2022.842964)
Supplement: Supplementary file 2 [file DataSheet1.docx]

**Dataset Links**

MEMO is available as a python package and can be found on https://github.com/mandelbrot-project/memo. It can also be installed through <https://pypi.org/project/memo-ms/>.

For the evaluation dataset, data and methods have been deposited on the GNPS/MASSive repository under accession number MSV000083306 at: <https://massive.ucsd.edu/ProteoSAFe/dataset.jsp?task=c5312f8ecb7d45feb35a723ba68874d2>

The GNPS Feature-Based Molecular Networking (FBMN) job is available at <https://gnps.ucsd.edu/ProteoSAFe/status.jsp?task=044e981ff0d84246ae5c91ef3db643a8>

The GNPS Qemistree job is available at <https://gnps.ucsd.edu/ProteoSAFe/status.jsp?task=8ca56d6e33bc4106b46ba5e3510c91cb>

For the plant extract dataset, raw and processed data have been deposited on the GNPS/MASSive repository under accession number MSV000087728, available at: <https://massive.ucsd.edu/ProteoSAFe/dataset.jsp?accession=MSV000087728>

The samples' metadata are also available on the MASSive repository at <https://massive.ucsd.edu/ProteoSAFe/dataset_files.jsp?task=b753bf1e39cb4875bdf3b786e747bc15#%7B%22table_sort_history%22%3A%22main.collection_dsc%22%2C%22main.attachment_input%22%3A%22updates%2F2021-12-21_pmallard_cde57c76%22%2C%22main.collection_input%22%3A%22metadata%7C%7CEXACT%22%7D>

For the Waltheria indica samples, raw and processed data have been deposited on the GNPS/MASSive repository accession number MSV000088521, available at

<https://massive.ucsd.edu/ProteoSAFe/dataset.jsp?task=81caae4ac3724aacafaeab45ed989157>

All scripts used for data analysis are available at <https://github.com/mandelbrotproject/memo_publication_examples>.
